# Tumor-infiltrating immune cell signature score reveals prognostic biomarkers and therapeutic targets for colorectal cancer

**DOI:** 10.3389/fimmu.2025.1583327

**Published:** 2025-05-14

**Authors:** Xiaofei Zuo, Wujun Long, Kai Lin, Guiqing Jia

**Affiliations:** ^1^ Department of Gastrointestinal Surgery, Sichuan Provincial People’s Hospital, School of Medicine, University of Electronic Science and Technology of China, Chengdu, China; ^2^v Department of Gastrointestinal Surgery, Sichuan Provincial People’s Hospital, University of Electronic Science and Technology of China, Chengdu, China

**Keywords:** colorectal cancer, tumor-infiltrating immune cells, immunotherapy response prediction, chromosomal instability, TIIC-RNAs

## Abstract

**Background:**

Colorectal cancer (CRC) is one of the leading contributors to cancer-related deaths worldwide, with more than 900,000 new diagnoses and related deaths each year. This study aims to explore the prognostic value of tumor-infiltrating immune cell (TIIC)-related genes in CRC, in order to discover new biomarkers and therapeutic targets.

**Methods:**

We integrated CRC transcriptome data from public databases to construct and validate a prognostic model and analyzed single-cell RNA sequencing (scRNA-seq) data to classify immune cell subtypes. A suite of computational models was employed to assess TIIC signature scores and to refine the selection of prognostic TIIC-related genes using multiple machine learning techniques—including Random Survival Forest (RSF), LASSO regression, and Cox proportional hazards regression, among others. In addition, pathway enrichment, immune signature difference analyses, and immunotherapy response predictions were performed. Potential biomarkers and therapeutic targets were identified through differential gene analysis, gene set enrichment analysis (GSEA), and copy number variation (CNV) landscape comparisons between high and low TIIC groups.

**Results:**

We identified 137 significant TIIC-RNAs within the CRC microenvironment and developed a prognostic model based on five key TIIC-RNAs. This model, which leveraged machine learning methods such as RSF, LASSO, and Cox regression, demonstrated outstanding performance in survival prediction across TCGA-CRC and external validation datasets, outperforming 22 existing prognostic models. Furthermore, the high TIIC score group showed heightened expression of angiogenesis-related genes, whereas the low score group was enriched for immune response-associated genes. The TIIC signature score was significantly correlated with tumor-infiltrating immune cells, various metabolic characteristics, and chromosomal instability, and it effectively predicted immunotherapy response across diverse cancer types.

**Conclusion:**

The findings of this study highlighted the promise of the TIIC signature score in forecasting the outcomes for CRC patients. Additionally, it emphasized its utility in predicting the effects of immunotherapy, thereby enhancing our comprehension of the intricacies within the tumor microenvironment. Further research needs to concentrate on assessing the clinical utility of the TIIC signature score while also confirming its relevance across various populations and treatment contexts.

## 1 Introduction

Colorectal cancer (CRC) is considered one of the most lethal cancers worldwide, with around 1.2 million new cases and 600,000 deaths each year (1). The development of CRC is linked to numerous factors, including age, genetic predisposition, chronic inflammatory bowel conditions, and poor lifestyle and dietary choices (2). CRC poses significant health and economic challenges, not only threatening health, but also bringing huge economic pressure. The management of CRC typically entails a multifaceted approach, incorporating various techniques like surgical procedures, chemotherapy, radiotherapy, and targeted drug therapies (3). Despite notable progress in treatment technologies, the five-year survival rate for CRC continues to be comparatively low, especially when the disease is identified at a later stage (4). The limitations of treatment options and the uncertainty of treatment effects have presented significant challenges for clinicians and patients. In the face of CRC, the main dilemma in the current treatment field is how to improve the effectiveness of treatment, reduce side effects, and achieve personalized medicine (5). At present, the lack of knowledge about CRC biomarkers limits the precise stratification of patients and the choice of treatment paths. Simultaneously, the intricate nature of the CRC tumor microenvironment makes it challenging for current therapies to fully address the drug resistance and immune evasion exhibited by tumor cells.

The role of tumor-infiltrating immune cells (TIICs) in CRC is gaining increasing attention (6–8). The TIIC signature score is an emerging bioinformatics tool that combines scRNA-seq data to quantify the presence and activity of TIICs. This score can help identify the patient population most likely to respond to immunotherapy and provide new insights into the biological behavior of CRC. In recent years, the TIIC signature score has been applied in multiple clinical studies, especially in predicting the efficacy of immunotherapy, with encouraging results (9). For example, in breast cancer, lung cancer, and other types of solid tumors, the TIIC signature score has been shown to effectively help distinguish patients with good prognosis from those with poor prognosis (10).

This research emphasizes the predictive significance of genes associated with TIICs in CRC. By integrating data from the Gene Expression Omnibus (GEO) and The Cancer Genome Atlas (TCGA), we developed an innovative scoring system centered on a TIIC signature that successfully predicts the survival outcomes of CRC patients within multiple validation datasets. The TIIC signature score excels beyond conventional clinical metrics and demonstrates strong effectiveness in forecasting the success of immunotherapy. Our study provides better insights into the design of personalized treatment options for CRC patients.

## 2 Materials and methods

### 2.1 Cell culture

Colorectal cancer (CRC) cell lines LoVo and SW480, along with normal colorectal epithelial cell line NCM460, were purchased from Shanghai Zhongqiao Xinzhuo Biotechnology Co., Ltd. and ATCC (Manassas, VA, USA), respectively. All cell lines were cultured in DMEM medium (Solarbio, Beijing, China) supplemented with 10% fetal bovine serum (FBS) and 1% penicillin-streptomycin. Cells were maintained at 37°C in a 5% CO_2_ incubator.

### 2.2 RNA extraction and quantitative PCR

Total RNA was isolated from the cultured cells using TRIzol reagent (Invitrogen, Carlsbad, CA, USA) following the manufacturer’s protocol. RNA quality and concentration were assessed using a NanoDrop spectrophotometer. cDNA was synthesized from 1 μg of RNA using ReverTra Ace qPCR RT Premix (Toyobo, Osaka, Japan) and the gDNA Remover Kit (Toyobo, Osaka, Japan). Reverse transcription was carried out at 42°C for 60 minutes, followed by enzyme inactivation at 95°C for 5 minutes. Quantitative real-time PCR (qRT-PCR) was performed using SYBR Premix Ex Taq II (Takara Bio, Japan) on the Mx3005P real-time PCR system (Stratagene, San Diego, CA, USA). GAPDH was used as the endogenous control. The following thermal cycling conditions were used: initial denaturation at 95°C for 10 minutes, followed by 45 cycles of 95°C for 5 seconds, 60°C for 30 seconds. The relative expression levels of the target genes were calculated using the 2^(-ΔΔCt) method, with each sample analyzed in triplicate. Primer sequences are provided in **Supplementary Table 1**.

### 2.3 Acquisition and processing of transcriptome data

This research utilized RNA expression profiles along with matching clinical data (n=606) related to colorectal cancer sourced from the public repository TCGA (https://portal.gdc.cancer.gov/) to create the training set for the model (11). The data underwent conversion into Transcripts Per Million (TPM) format, which was subsequently log2 transformed to enable more detailed analysis. Simultaneously, a validation set was created using a dataset comprising over 50 samples sourced from GEO (https://www.ncbi.nlm.nih.gov/geo/), which included colorectal cancer chip data from GSE12945 (n=62), GSE17537 (n=55), and GSE39582 (n=579) (12). To adjust the chip data, the normalizeBetweenArrays function from the limma package was utilized. TCGA and GEO are open-source databases and do not require additional ethical approval. We follow the regulations for data acquisition and use.

### 2.4 Acquisition and processing of scRNA-seq data

The single-cell dataset employed in this research comes from GSE166555, which is available in the GEO database and includes 13 CRC tumor specimens. For the data analysis, we applied the Seurat package. To maintain cell quality, the mitochondrial content should remain under 10%. The acceptable ranges for the UMI count of cells and the gene count are between 200 and 20,000, and 200 to 5,000, correspondingly. We performed data normalization and identified the most variable genes, totaling 2,000. The Seurat package offers various functions aimed at transforming data, particularly to reduce the impact of the cell cycle, by setting the parameter vars.to.regress to c(“S.Score”, “G2M.Score”). To achieve this objective, the functions NormalizeData, FindVariableFeatures, and ScaleData are applied. Furthermore, the harmony package is used to manage batch effects. The dimensionality reduction technique known as t-distributed stochastic neighbor embedding (tSNE) is likewise derived from Seurat and is used for visualization (13).

We employ a range of markers tailored for various cell types. For epithelial cells, we use markers such as “EPCAM,” “KRT18,” “KRT19,” and “CDH1.” For fibroblasts, the relevant markers include “DCN,” “THY1,” “COL1A1,” and “COL1A2.” In the case of endothelial cells, we identify markers like “PECAM1,” “CLDN5,” “FLT1,” and “RAMP2.” T cell markers that we utilize are “CD3D,” “CD3E,” “CD3G,” and “TRAC.” Furthermore, for NK cells, we recognize markers such as “NKG7,” “GNLY,” “NCAM1,” and “KLRD1.” B cells are characterized by markers including “CD79A,” “IGHM,” “IGHG3,” and “IGHA2.” For myeloid cells, the markers we consider are “LYZ,” “MARCO,” “CD68,” and “FCGR3A.” Lastly, the markers that denote mast cells are “KIT,” “MS4A2,” and “GATA2.” Separate annotations were made for different cell subgroups. Using these annotations, we created TSNE diagrams and violin plots to visualize cell markers, among other visual representations. A volcano plot depicted the genes that showed differential expression between immune cells and CRC cells. Following this, all immune cells were categorized into a single cluster, whereas the tumor cells were assigned to a distinct cluster, with automatic annotation conducted using Sc-Type software. In the end, we utilized the FindAllMarkers function to pinpoint the genes with differential expression when comparing immune cells to CRC cells. This analysis was conducted with parameters that encompassed a p-value below 0.05, a |log2FC| greater than 0.25, and an expression ratio exceeding 0.1.

### 2.5 Obtaining TIIC-related genes

Through a thorough examination of immune and tumor cells using single-cell RNA sequencing, along with an analysis of colorectal cancer tissues via bulk sequencing, a computational framework employing various algorithms was created. This framework aims to determine TIIC feature scores and to select the most significant TIIC-related RNAs. The detailed process proceeds as follows:

1. From the expression values, the RNAs in the top 15% were selected as candidates for immune-related RNAs.
2. Tissue-specific index (TSI) was used to identify potential immune-related RNAs:
$${\text{TSI}}_{\text{RNA}}=\frac{\sum_{i=1}^{N}\left(1-x_{RNA,i}\right)}{N-1}$$
  In this framework, N represents the number of different types of immune cells and xRNAs, while i signifies the expression level of RNA in the i-th immune cell, which is established by the normalized peak expression of RNA throughout all cell types. TSI ranges from 0 to 1; an RNA is considered universal to immune cells when TSI is 0, and when TSI reaches 1, it is classified as specific to a particular immune cell. Those RNAs that exhibit elevated expression across all types of immune cells are termed immune-related universal RNAs (iuRNAs).
3. TIIC-RNAs are characterized by a notable increase in immune cell types while exhibiting a decrease in tumor cells’ expression levels.
4. To enhance classification, a range of machine learning techniques such as Boruta, extreme gradient boosting (Xgboost), least absolute shrinkage and selection operator (LassoLR), random forest (RF), and microarray prediction analysis (Pamr) were employed, with the overlapping outcomes utilized to identify the most significant TIIC-RNA.

### 2.6 Construction and evaluation of TIIC prognostic model

In order to explore and demonstrate the prognostic relevance of Tumor-Infiltrating Immune Cells RNA (TIIC-RNA) in relation to overall survival in colorectal cancer patients drawn from the TCGA cohort, we first undertook a univariate Cox proportional hazards regression analysis. This statistical method allowed us to determine the relationship between TIIC-RNA profiles and patient survival rates, highlighting the importance of immune cell gene expression in predicting outcomes for individuals with colorectal cancer. Subsequently, we applied three distinct machine learning algorithms specifically designed for survival analysis—LassoCox, CoxBoost, and random forest. These approaches were chosen for their ability to effectively handle and analyze complex datasets, permitting a deeper investigation into the significance of TIIC-RNA in survival assessments. By leveraging these advanced analytical techniques, we aimed to enhance our understanding of how TIIC-RNA can serve as a valuable predictor in clinical settings. Moreover, we expanded our analysis by implementing a total of 20 different machine learning algorithms for scoring purposes. This diverse array of methodologies included Random Survival Forest (RSF), Conditional Random Forest (CForest), Lasso-Cox, Elastic Net Regression (Enet), Ridge Regression, Gradient Boosting based on Regression Trees (BlackBoost), Parametric Survival Model Regression (SurvReg), Conditional Inference Trees (CTree), Cox Proportional Hazards (CoxPH), Oblique Random Survival Forest (ObliqueRSF), Stepwise Cox Regression (StepwiseCox), Survival Support Vector Machine (SurvivalSVM), Generalized Boosted Regression Model (GBM), Ranger, Partial Least Squares Regression with Cox Model (PlsRcox), Gradient Boosting using Generalized Linear Model (GlmBoost), Supervised Principal Components (SuperPC), Akritas Conditional Nonparametric Survival Estimator (Akritas), and CoxBoost. The inclusion of these varied algorithms allowed for robust comparisons and strengthened our findings regarding the prognostic implications of TIIC-RNA. We then identified the most reliable model based on the comprehensive C-index derived from the external validation dataset. Based on the median TIIC values for each cohort, patients were categorized into groups with high and low TIIC scores. We examined the survival outcomes for these two groups in both TCGA-CRC and external validation datasets. Thereafter, ROC curves were created to evaluate the predictive capabilities of the models. Additionally, utilizing the TCGA dataset, we demonstrated the differences in survival outcomes, tumor stages, and TNM classifications between the two categories of TIIC signature scores, along with the C-index variations for various evaluation metrics across each dataset. To improve the assessment of the prognostic effectiveness of the TIIC signature score, we included 22 prognostic models reported in the literature and compared their C-index results with that of the TIIC signature score across TCGA-CRC and other validation datasets (14–35).

### 2.7 Analysis of pathway enrichment and immune characteristics between the two TIIC groups

To begin with, we examined the biological traits of the two TIIC signature score groups through Gene Set Variation Analysis (GSVA) analysis sourced from the MsigDB database (36). Following this, we demonstrated the variations in Gene Ontology (GO) and Kyoto Encyclopedia of Genes and Genomes (KEGG) pathway activities among the two TIIC signature score groups using t-SNE plots (13, 37, 38). In addition, we carried out enrichment analysis and visualization of the differentially expressed genes that set apart the TIIC score groups, drawing upon data from Metascape. Finally, we performed GSEA analysis on the differentially expressed genes differentiating the two score groups, employing the GO pathway list.

The TIMER algorithm, known for estimating tumor immune response, was utilized to analyze six types of immune cells. Simultaneously, we applied the single-cell gene set enrichment analysis (ssGSEA) method for evaluating 28 immune cell types. Moreover, the Microenvironment Cell Populations-counter (MCPcounter) algorithm was used to assess 10 categories of immune cells. Lastly, the Estimation of Cell Populations and Immune Cells in Tumors Using Expression Data (ESTIMATE) algorithm facilitated the assessment of immune-infiltrating cell presence, which we represented using heat maps. In addition, we compared the differences in mRNA expression, methylation level, CNV amplification, and CNV deep deletion of immune checkpoint-related genes between the 2 groups.

### 2.8 Metabolic signature prediction related to immunotherapy response and TIIC signature score

The evaluation of the response to immunotherapy was conducted using a comprehensive array of data from multiple sources, specifically focusing on different cancer types. The datasets included studies related to melanoma such as those from Nathanson and GSE35640, as well as GSE91061 and GSE78220, all of which further contributed to our understanding of melanoma responses. Additionally, we incorporated data from IMvigor210, which pertains to urothelial carcinoma (UC), and research from Braun that relates to renal cell carcinoma (RCC). Further expanding our analysis, we utilized datasets GSE179351 that examines both colorectal adenocarcinoma and pancreatic adenocarcinoma (COAD and PAAD), GSE165252 focused on esophageal adenocarcinoma (ESCA), GSE103668 concerning triple-negative breast cancer (TNBC), and GSE126044, which pertains to non-small cell lung cancer (NSCLC). To assess the immunotherapy response across these various datasets, we calculated the TIIC signature scores for each dataset. This process allowed us to effectively evaluate the underlying immune dynamics associated with treatment responses. Furthermore, we extended our analysis to the TCGA dataset by employing the TIDE online platform, available at http://tide.dfci.harvard.edu/. This platform enabled us to perform a thorough analysis of the immune response and scoring, further enriching our understanding of how different cancer types respond to immunotherapy initiatives.

Additionally, to investigate the overall metabolic traits in the two groups identified by the TIIC signature scores, we performed GSVA on metabolic pathways found in the KEGG database and assessed the correlation between the TIIC signature scores and the respective metabolic pathways.

### 2.9 Comparison of genomic variation landscapes between the two groups

The R package “maftools” was employed to analyze the mutation data and explore the variations in mutation load across the two groups. The waterfall plot, displaying the top 30 genes, was generated for both high-risk and low-risk groups using maftools. To evaluate the differing mutation frequencies of genes in these two groups, a chi-square test was performed. CNV data were analyzed with Gistic 2.0 software. Following this, chromosome segments that showed significant amplification and deletion were detected, and variations in CNVs across chromosomes were assessed. Moreover, we calculated the fraction of genomic alterations (FGA), the fraction of gained genomes (FGG), and the fraction of lost genomes (FGL). Ultimately, the findings related to CNV were illustrated using the R package “ggplot2.”

### 2.10 Methodological rationale and data processing details

1. Justification of Machine Learning Models for Prognostic Analysis
  To construct a robust prognostic model, we employed multiple machine learning algorithms—including CoxBoost, LassoCox, and Random Survival Forest (RSF)—because they are well established for handling high-dimensional genomic data, offer robust variable selection, and minimize overfitting risks. In addition, by evaluating a comprehensive set of 20 algorithms, we ensured that the final model—selected based on the highest concordance index (C-index) in external validation cohorts—was both reliable and reproducible.
2. Criteria for Selecting the Top 15% RNA Candidates
  From the expression profiles of immune cell populations, the top 15% of RNAs were selected as candidate immune-related transcripts. This threshold was determined empirically, as preliminary analyses indicated that this cutoff efficiently captures the most highly expressed—and thus potentially the most biologically relevant—RNAs while reducing background noise. Similar selection criteria have been employed in previous studies to identify key immune markers.
3. Batch Effect Handling in Transcriptomic Data Normalization
  To minimize technical variability and correct for batch effects, we applied tailored normalization strategies across our datasets. For TCGA RNA-seq data, expression values were converted to Transcripts Per Million (TPM) and log2-transformed for standardization. For GEO microarray datasets, we used the normalizeBetweenArrays function from the limma package to adjust for inter-array differences. For single-cell RNA-seq data, batch effects were addressed using the Harmony package, which integrates data from multiple samples while preserving true biological variability.

### 2.11 Statistical analysis

Data processing, statistical evaluation, and graphing were analyzed with R version 4.1.3. The Pearson correlation coefficient was employed to evaluate the relationship between two continuous variables. For the categorical variables, the chi-square test was used for comparisons, whereas continuous variables were examined using either the T-test or the Wilcoxon rank-sum test. The survminer package was utilized to establish the optimal cutoff value. Kaplan-Meier analyses and Cox regression assessments were performed using the survival package. A p-value below 0.05 was considered to indicate statistical significance (* p < 0.05; ** p < 0.01; *** p < 0.001; **** p < 0.0001).

## 3 Results

### 3.1 Identification of TIIC-RNAs at the single-cell level

**Figure 1:**
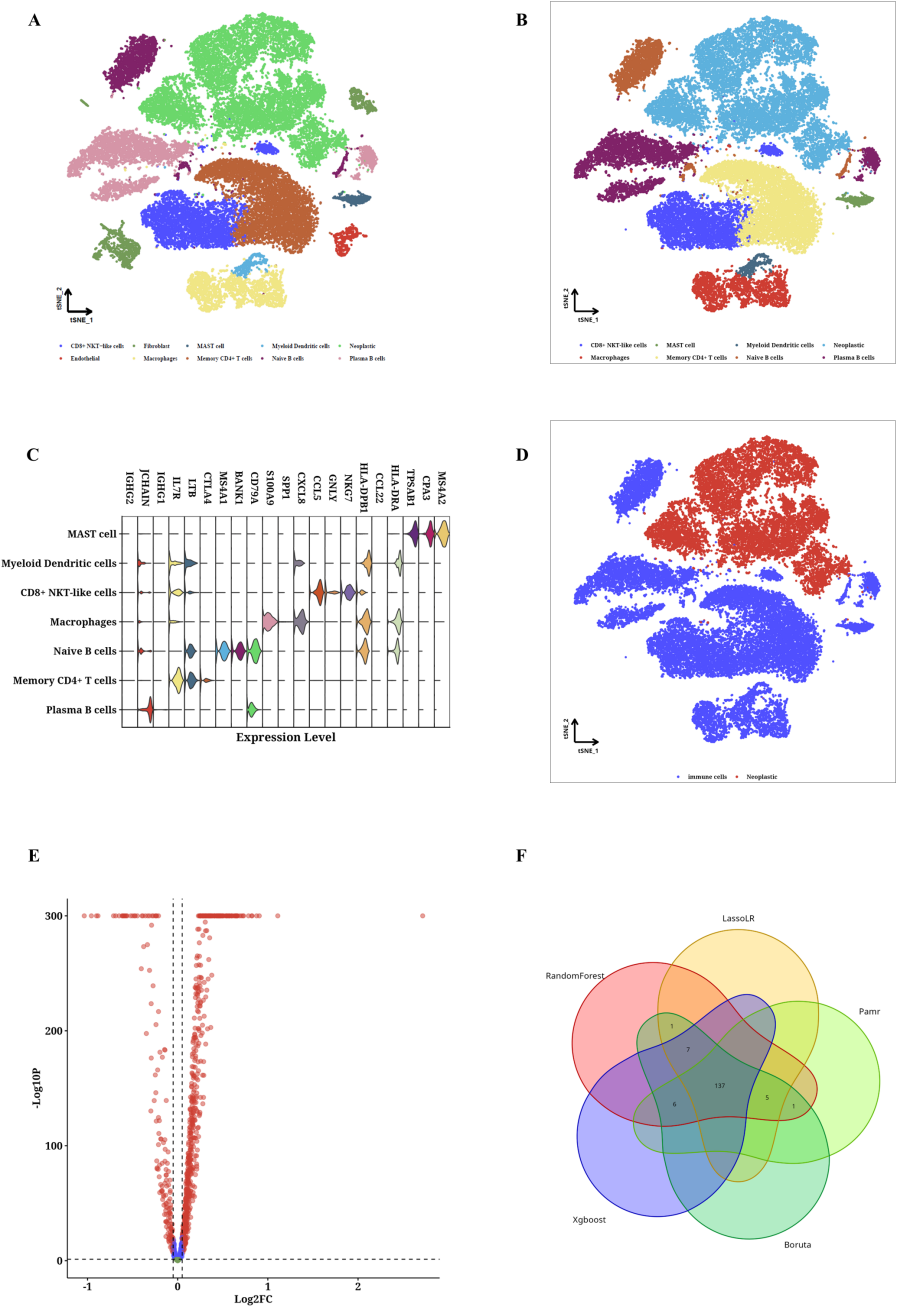
TIIC-RNA results were identified at the single-cell level. **(A)** t-SNE maps of identified microenvironment cells and CRC cells. **(B)** t-SNE diagrams of identified CRC cells and 4 types of immune cells. **(C)** Violinplot of differentially expressed genes in identified immune cells. **(D)** t-SNE maps of identified immune cells and CRC cells. **(E)** Volcano map of differentially expressed genes between immune cells and CRC cells. **(F)** Venn diagram shows the classification by crossing genes identified by five ML algorithms.

By employing the CRC scRNA-seq dataset, we identified CRC cells along with nine varieties of microenvironment cells (see **Figure 1A**). For subsequent analysis, CRC cells alongside seven immune cell types were chosen (see **Figure 1B**). We determined that the highest 15% of RNA molecules exhibited expression in each type of immune cell, amounting to a total of 4,743 RNAs considered as potential immune-related RNAs. Utilizing the TSI score threshold (TSI < 0.35), we identified 1,009 of these immune-related RNAs as IURNAs. **Figure 1C** presents the differentially expressed genes (DEGs) found within immune cells to emphasize the precision of the identified cell types. The tSNE plots showing immune cells in relation to CRC cells are depicted in **Figure 1D**, where we performed a screening of differentially expressed genes (DEGs) between these cell types, as illustrated in **Figure 1E**. Compared to CRC cells, 157 DEGs that were significantly upregulated in immune cells were categorized as TIIC-RNAs. Leveraging the previously identified TIIC-RNAs, we applied five machine learning algorithms for classification: Boruta, Xgboost, LassoLR, RF, and Pamr. This approach allowed us to identify the 137 most important TIIC-RNAs, as demonstrated in **Figure 1F**. Correspondingly, we validated the expression levels of AIP, HNRNPH1, UBE2D2, and NFKB2 in CRC cell lines through quantitative PCR. The results showed a significant reduction in the expression of AIP and HNRNPH1, while UBE2D2 and NFKB2 expression were significantly elevated (**Supplementary Figure 1**). To ensure the robustness of our analysis, we performed cross-validation using independent datasets. In addition to constructing the prognostic model with the TCGA-CRC cohort, we validated the TIIC signature score in multiple independent GEO datasets (GSE12945, GSE17537, and GSE39582). The consistent performance across these datasets demonstrates the model’s stability and supports its potential clinical applicability.

### 3.2 Construction of TIIC prognostic model

**Figure 2:**
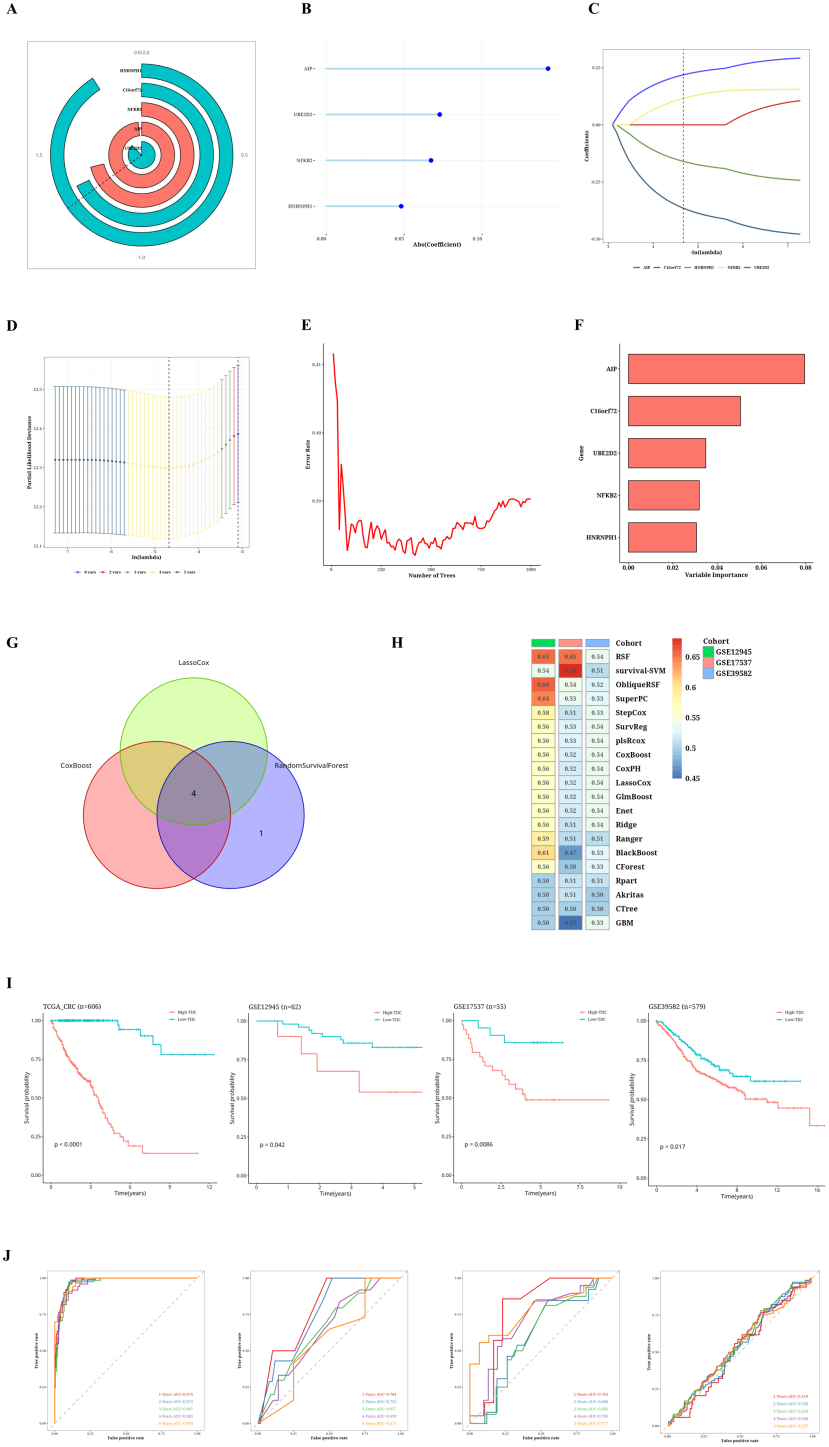
Results of TIIC prognostic model construction. **(A)** Univariate Cox regression analysis of TIIC-RNA. **(B)** Reduce the dimension of prognostic genes by CoxBoost algorithm. **(C, D)** Reduce the dimension of prognostic genes by LassoCox algorithm. **(E, F)** Dimension reduction of prognostic genes was carried out by random survival forest algorithm. **(G)** Venn diagram shows intersecting survival prognostic genes identified by three ML algorithms. **(H)** Score using 20 ML algorithms, based on a comprehensive C-index situation of externally validated datasets. **(I)** Kaplan-Meier survival curves for TIIC signature scores for OS in TCGA-CRC and other validation datasets. **(J)** Time-dependent ROC curves of the TIIC signature score for 1-5 years of OS in TCGA-CRC and other validation datasets.

A Cox proportional hazards regression analysis focusing on a single variable was performed to evaluate the prognostic importance of TIIC-RNA concerning overall survival (OS) in individuals diagnosed with CRC. In the TCGA dataset, five TIIC-RNAs were identified (**Figure 2A**). Subsequently, three distinct machine learning (ML) methods for conducting survival analysis were applied to these five genes: CoxBoost (**Figure 2B**), LassoCox (**Figures 2C, D**), and random forest (**Figures 2E, F**). By cross-referencing the results from these three techniques, four prognostic TIIC-RNAs were identified (**Figure 2G**). Following this, a comprehensive set of 20 ML algorithms was utilized to create the prognostic model, with the most dependable model being derived from the overall C index of the external validation dataset. Among these 20 ML algorithms, the RSF algorithm demonstrated the best performance (see **Figure 2H**). In the TCGA-CRC cohort, patients with higher TIIC signature scores experienced significantly poorer survival outcomes (p < 0.05; see **Figure 2I**). The predictive performance of the TIIC signature score was robust, as demonstrated by the time-dependent ROC curves, which yielded AUC values of 0.975, 0.972, 0.965, 0.965, and 0.975 for 1-, 2-, 3-, 4-, and 5-year overall survival predictions, respectively (**Figure 2J**). To further evaluate its prognostic value, we compared our model with 22 previously published prognostic models using standard evaluation metrics, including the concordance index (C-index) and AUC. Our TIIC signature score achieved a C-index close to 0.97, which was consistently higher than the values reported for the other models (most of which were below 0.95), thereby demonstrating its superior predictive accuracy across both the TCGA-CRC dataset and external validation cohorts. The AUC values for the other datasets are displayed in the figure (**Figure 2J**). Moreover, the robustness of the TIIC signature score was further confirmed in independent GEO validation cohorts. The model consistently demonstrated high predictive accuracy across different datasets, thereby reinforcing its potential as a reliable tool for clinical risk stratification and personalized treatment decision-making.

### 3.3 Comparison of the prognostic value between TIIC signature score and previous features

**Figure 3:**
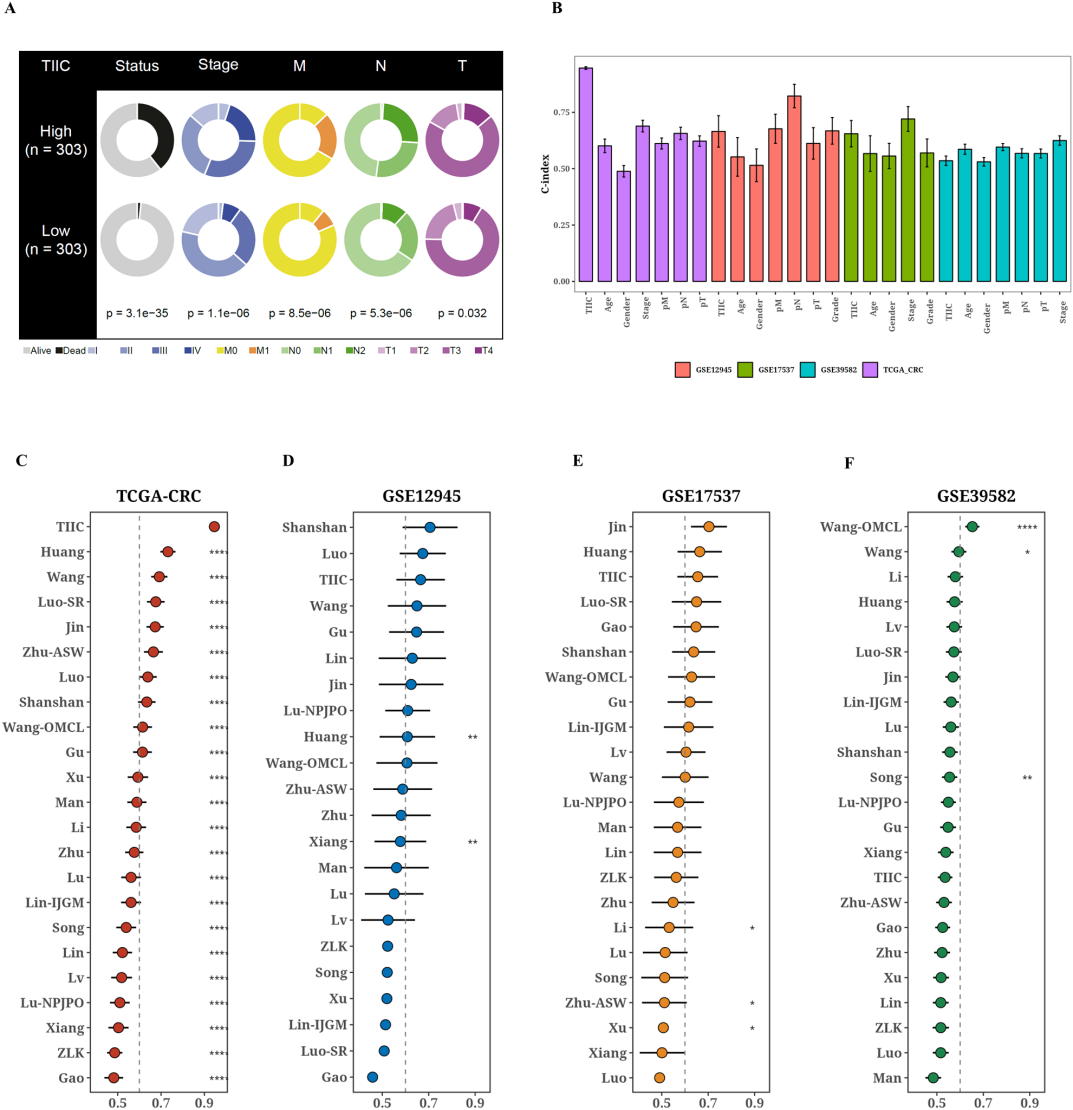
Comparison of prognostic value between TIIC signature score and previous features. **(A)** Comparison of different clinical factors in the two TIIC signature assessment groups. **(B)** TCGA-CRC and other validation datasets with TIIC feature scores and C-index bars for various clinical factors. **(C–G)** TCGA-CRC and other validation datasets TIIC feature scores and C-index plots of 22 colorectal cancer models.

The TCGA dataset revealed notable variations in survival outcomes, tumor stage, and the TNM staging system among the two TIIC signature score groups (p < 0.05, **Figure 3A**). The group identified as high-risk exhibited a more advanced disease progression and increased mortality rates. Moreover, the TIIC signature score demonstrated superior performance in C-index across various datasets when compared to factors such as age, gender, tumor stage, and the TNM staging system. This indicates a higher predictive efficacy than that of the conventional clinical feature system (**Figure 3B**). To evaluate the prognostic effectiveness of the TIIC signature score in relation to other assessment systems, we integrated 22 prognostic models identified in the current literature and examined the C-index for each prognostic evaluation system across TCGA-CRC and diverse validation datasets (**Figure 3C–F**). Our TIIC model demonstrated superior performance compared to the majority of other previously published models in TCGA-CRC and external validation datasets.

### 3.4 Prediction of biological mechanisms associated with TIIC signature scores

**Figure 4:**
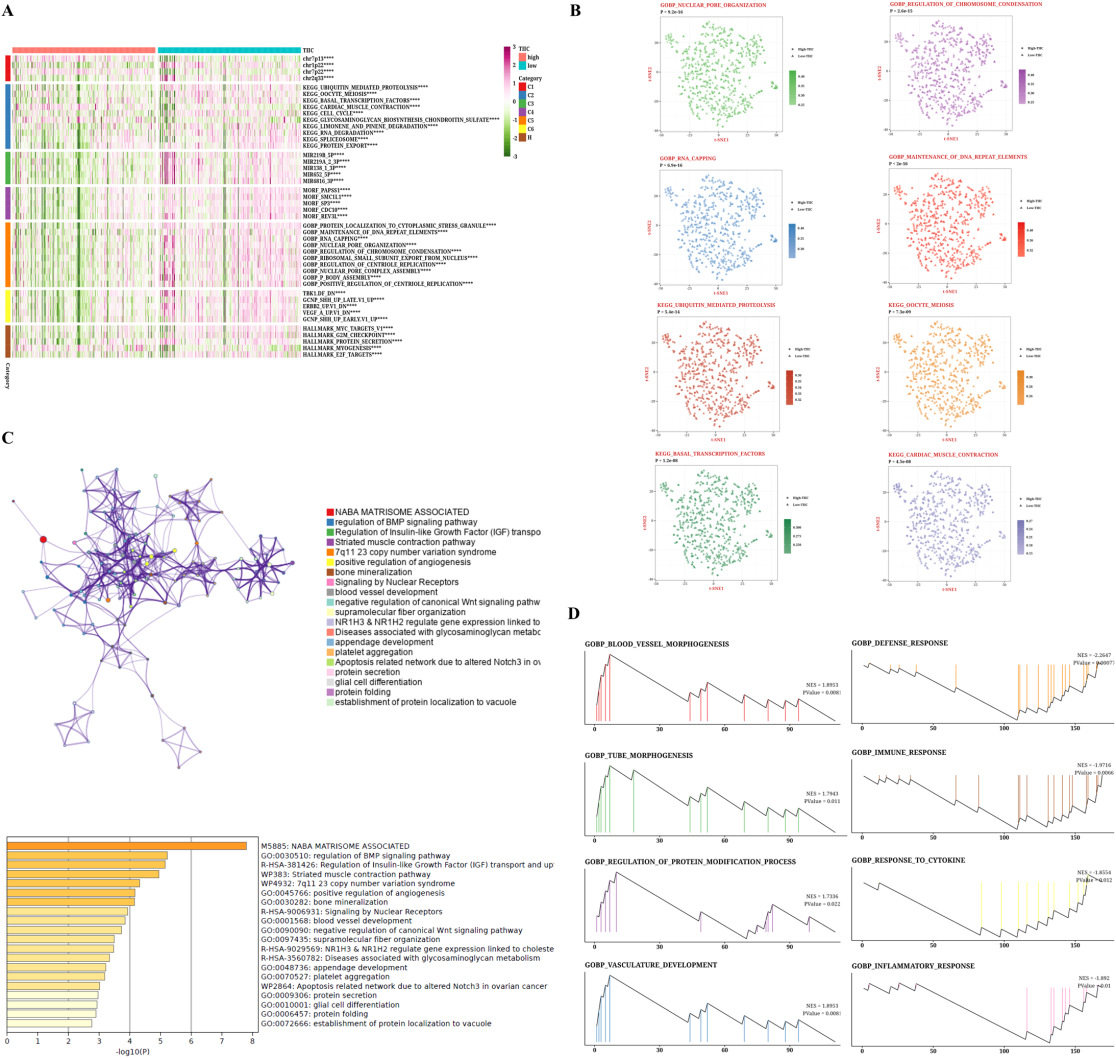
Biological characteristics of TIIC signature score in TCGA dataset. **(A)** GSVA analysis based on MsigDB describes the biological properties of the two TIIC feature assessment groups. **(B)** t-SNE plots for GO and KEGG describe the differences in path activity between the two TIIC signature rating groups. **(C)** Enrichment analysis of differentially expressed genes between groups with high TIIC rating based on Metascape. **(D)** GSEA result graphs of GO and KEGG for groups with high TIIC rating and groups with low TIIC rating.

Given the immune-related traits that are heightened in the low group, we intend to investigate the potential biological mechanisms in greater depth. Most pathways linked to a high TIIC signature score were reduced in comparison to those observed in the low group (**Figure 4A**). From the GOBP and KEGG databases, eight pathways were selected that showed notable differences between the two groups. The tSNE plots for these samples were presented, along with the associated ssGSEA scores for each pathway (**Figure 4B**). Furthermore, we demonstrated the enrichment outcomes for the upregulated genes within the high TIIC group through Metascape, revealing their connection to the BMP signaling pathway and the modulation of growth factors (**Figure 4C**). We presented the GSEA results for the prominent genes found in both the high TIIC group and the low TIIC group. The findings indicated that in the high TIIC group, there was an upregulation of GOBP_BLOOD_VESSEL_MORPHOGENESIS, GOBP_TUBE_MORPHOGENESIS, GOBP_REGULATION_OF_PROTEIN_MODIFICATION_PROCESS, and GOBP_VASCULATURE_DEVELOPMENT. In contrast, the low TIIC group exhibited upregulation of GOBP_DEFENSE_RESPONSE, GOBP_IMMUNE_RESPONSE, GOBP_RESPONSE_TO_CYTOKINE (noted twice), and GOBP_INFLAMMATORY_RESPONSE (see **Figure 4D**).

### 3.5 TIIC signature is significantly correlated with immune-related features

**Figure 5:**
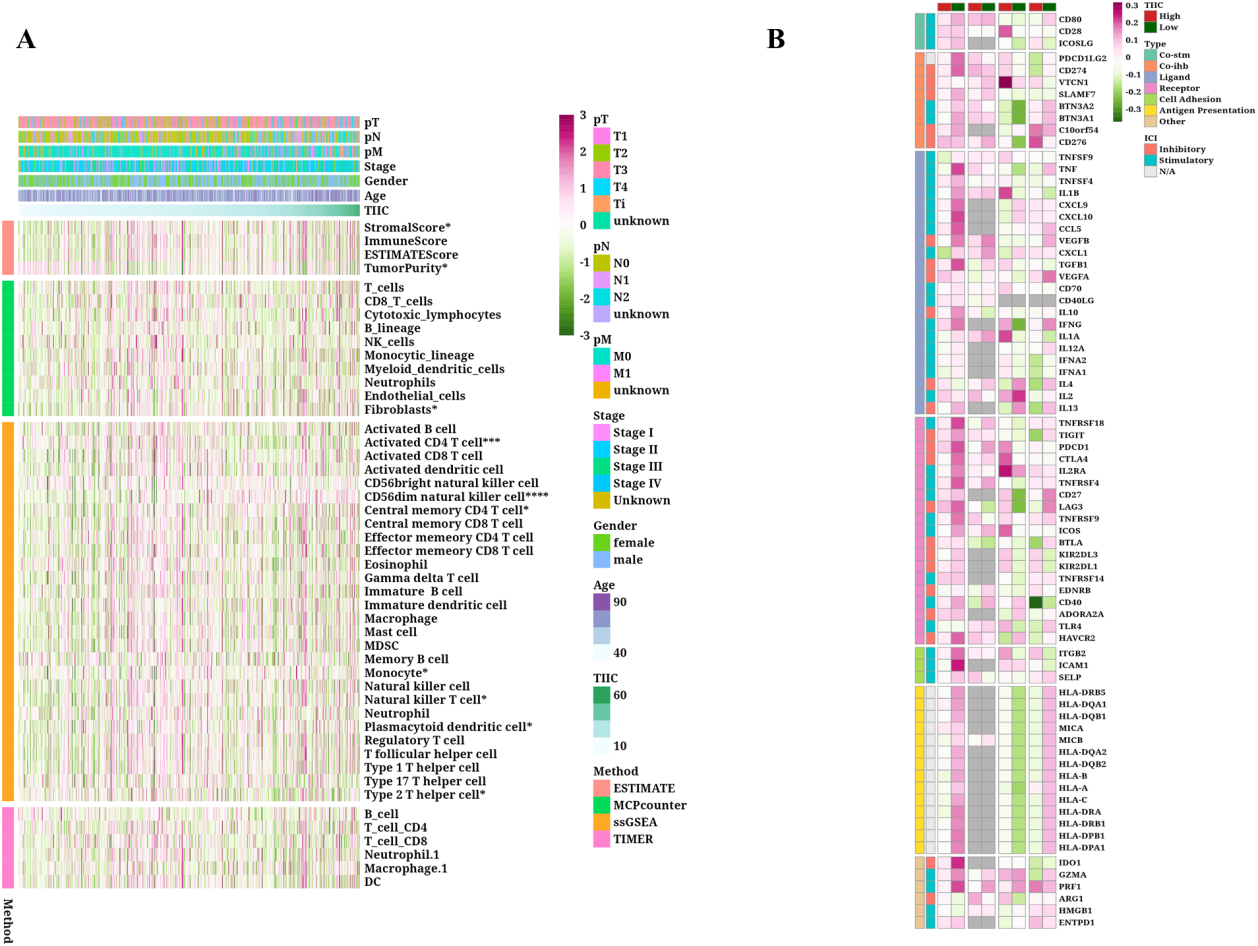
Immunological characteristics of TIIC signature score in TCGA dataset. **(A)** Correlation between feature scores and immunoinfiltrating cells. **(B)** Differences in mRNA expression, methylation degree, CNValue of Amp and CNvalue of Del between immunoregulatory genes in high and low TIIC groups.

The TIMER algorithm for tumor immune estimation was employed to assess six types of immune cells, whereas the Single Cell Gene Set Enrichment Analysis (ssGSEA) method was used for examining 28 different immune cell types. Furthermore, the MCPcounter algorithm was utilized to evaluate 10 different immune cell types, while the ESTIMATE algorithm was applied to estimate the number of immune infiltrating cells found within tumors. As the TIIC score rises, we observed an increase in the activity of the matrix score, TumorPurity, Fibroblasts, Activated CD4 T cells, CD56dim NK cells, and others (p < 0.05, **Figure 5A**). Additionally, we analyzed the TIIC score and compared the variations in mRNA expression, methylation level, CNValue of Amp, and CNValue of Del for each immune regulatory gene across the high and low TIIC groups (**Figure 5B**).

### 3.6 Validation of the predictive value of TIIC signature score for immunotherapy response in multiple datasets

**Figure 6:**
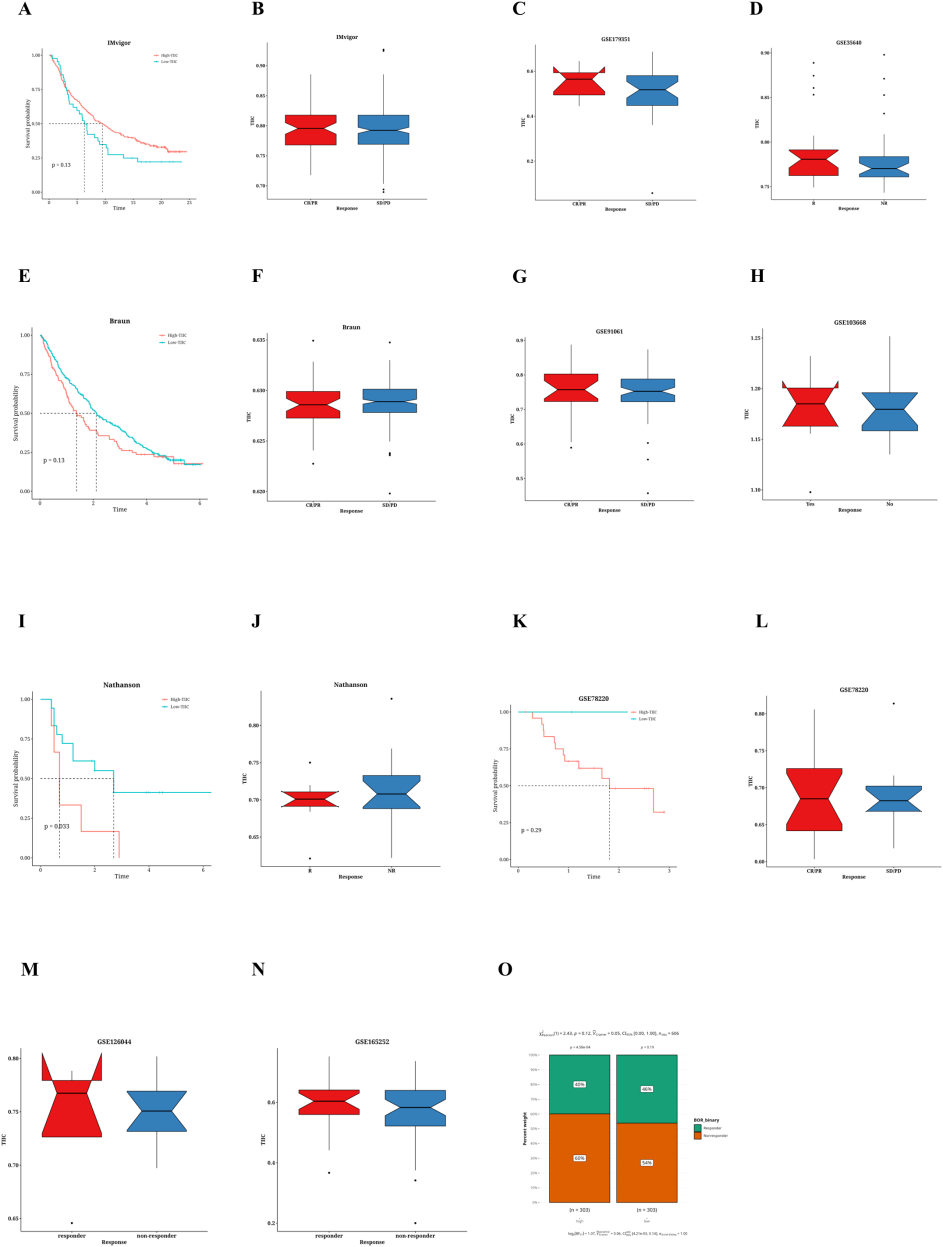
Prediction of immunotherapy response by TIIC characteristic score. **(A)** Kaplan-Meier survival curve for TIIC signature scores of OS in IMvigor dataset. **(B)** Association between TIIC feature scores and immunotherapy response in the Vigor dataset. **(C)** Association between TIIC signature scores and immunotherapy responses in the GSE179351 dataset. **(D)** TIIC signature score and immunotherapy response in the GSE35640 dataset. **(E)** Kaplan-Meier survival curve for TIIC signature scores of OS in Braun dataset. **(F)** Association between TIIC signature scores and immunotherapy response in the Braun dataset. **(G)** TIIC signature score and immunotherapy response in the GSE91061 dataset. **(H)** TIIC signature score and immunotherapy response in the GSE103668 dataset. **(I)** Kaplan-Meier survival curve for TIIC signature scores of OS in the Nathanson dataset. **(J)** Association between TIIC signature scores and immunotherapy response in the Nathanson dataset. **(K)** Kaplan-Meier survival curve for TIIC signature scores of OS in GSE78220 dataset. **(L)** Association between TIIC signature scores and immunotherapy response in the GSE78220 dataset. **(M)** TIIC signature score and immunotherapy response in the GSE126044 dataset. **(N)** TIIC signature score and immunotherapy response in the GSE165252 dataset. **(O)** The TIDE algorithm predicted the association between TIIC feature scores and immunotherapy responses in the TCGA dataset.

Taking into account the predictive potential of the TIIC signature score for the benefits of immunotherapy, we subsequently evaluated its efficacy using different immunotherapy datasets from various cancer types. Within the IMvigor dataset, patients with urothelial carcinoma who had elevated TIIC signature scores demonstrated improved survival rates (**Figure 6A**). Individuals diagnosed with ulcerative colitis (UC) who displayed a elevated tumor-infiltrating immune cell (TIIC) signature score showed a more favorable reaction to anti-PD-L1 immunotherapy (refer to **Figure 6B**). Moreover, an examination of the Braun dataset indicated that renal cell carcinoma (RCC) patients possessing a low TIIC signature score had improved survival rates (see **Figure 6E**). In the context of RCC patients, those exhibiting a high TIIC signature score demonstrated a superior response to anti-PD-1 immunotherapy (illustrated in **Figure 6F**). Furthermore, the Nathanson dataset suggested that patients with a low TIIC signature score also realized better survival outcomes (shown in **Figure 6I**). Patients presenting with a low TIIC signature score were observed to have a superior response to immunotherapy (as depicted in **Figure 6J**). In addition, the GSE78220 dataset revealed that individuals presenting with low TIIC signature scores had enhanced survival rates (as illustrated in **Figure 6K**) and exhibited a more positive response to immunotherapy (as indicated in **Figure 6L**). Moreover, patients who had high TIIC signature scores in the GSE179351 (COAD and PAAD) datasets (**Figure 6C**), GSE35640 (**Figure 6D**), GSE91061 (**Figure 6G**), GSE103668 (**Figure 6H**), GSE165252 (**Figure 6N**), and GSE126044 (**Figure 6M**) demonstrated a favorable immunotherapeutic response The TIDE algorithm indicated that within the TCGA dataset, the percentage of patients who responded was reduced in the group with a lower TIIC signature score (see **Figure 6O**).

### 3.7 Prediction of metabolic features associated with TIIC signature scores

**Figure 7:**
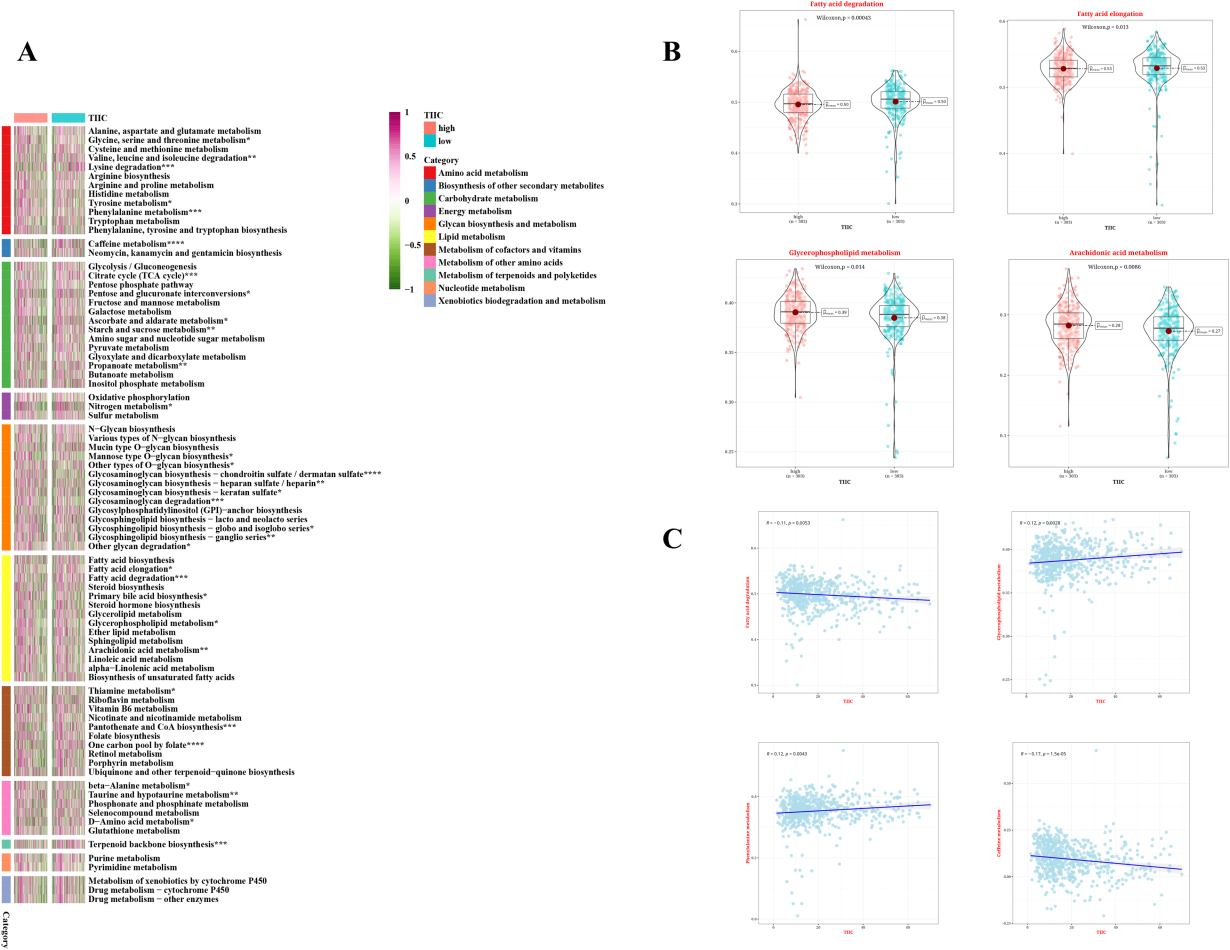
Metabolic characteristics of TIIC feature score in TCGA dataset. **(A)** The metabolic pathways of 11 metabolic classes in two TIIC characteristic assessment groups were analyzed based on KEGG GSVA. **(B)** Differences in metabolic pathways between the two TIIC profile groups. **(C)** Correlation between TIIC feature scores and KEGG-based GSVA analysis of metabolic pathways.

To investigate the comprehensive metabolic traits in the two groups identified by the TIIC signature score, a Gene Set Variation Analysis (GSVA) was performed on the metabolic pathways documented in the KEGG database. Furthermore, a notable correlation was found between the TIIC signature scores and various metabolic pathways (**Figure 7A**). Interestingly, the group with a low TIIC signature score exhibited significantly heightened activation of both fatty acid elongation and degradation processes. Conversely, the high TIIC signature score group demonstrated notably greater activation of Arachidonic acid metabolism and Glycerophospholipid metabolism (refer to **Figure 7B**). Furthermore, there was a negative correlation between the TIIC signature score and metabolic pathways including fatty acid degradation and caffeine metabolism, while a positive correlation with glycerophospholipid metabolism and phenylalanine metabolism was observed (p < 0.01, see **Figure 7C**).

### 3.8 SNV mutation difference analysis and CNV difference analysis

**Figure 8:**
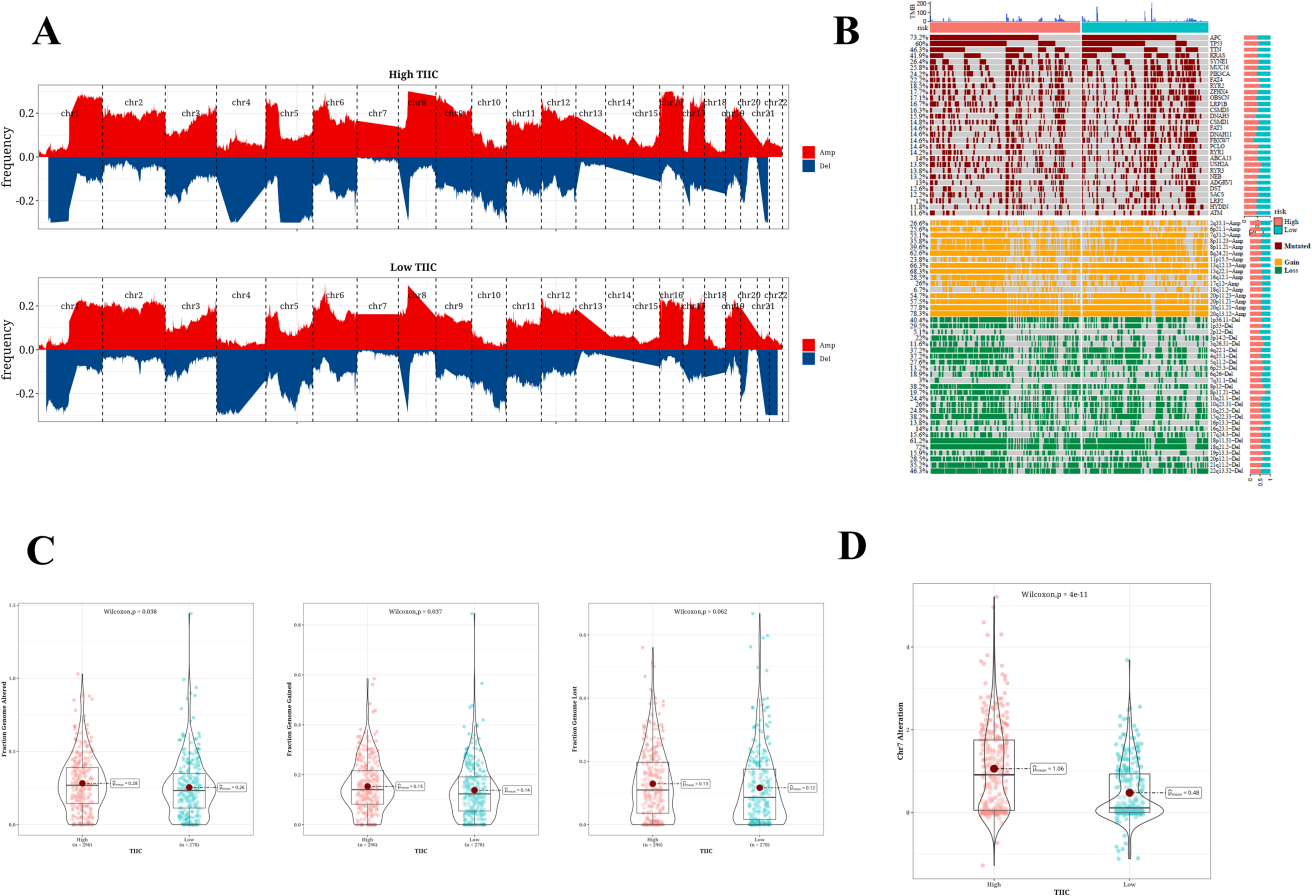
SNV mutation difference analysis and CNV difference analysis results. **(A)** Chromosome amplification and deletion based on GISTIC 2.0 in two TIIC signature assessment groups. **(B)** Genomic mutation landscape in two TIIC signature assessment groups. **(C)** Proportion of genome changes, portion of genome acquired, and proportion of genome loss in both TIIC signature assessment groups. **(D)** Distribution of CNV mutations in chr7 across two TIIC signature assessment groups.

Variations in the frequencies of chromosomal changes were observed between the two categories of TIIC signature scores (**Figure 8A**). The waterfall plot represents the modifications in the top 30 genes within these two risk categories. Clearly, APC (73.2%), TP53 (60%), and TTN (46.3%) show higher rates of mutation (**Figure 8B**). The cohort displaying a high score for the TIIC signature showed heightened chromosomal instability, as evidenced by FGA, FGG, and FGL. Significant statistical differences were noted in FGA and FGG, while FGL did not reveal any remarkable alterations (**Figure 8C**). The difference in CNV mutation on chromosome 7 was pronounced between the two groups (**Figure 8D**).

## 4 Discussion

CRC is one of the leading contributors to cancer-related deaths worldwide, causing approximately 1.2 million new cases and close to 600,000 deaths each year (39). Its occurrence is associated with factors such as age, genetics, chronic intestinal inflammation, unhealthy lifestyle and diet (40). CRC not only threatens health, but also brings a heavy economic burden. Treatment typically involves a combination of surgery, chemotherapy, radiotherapy, and targeted therapy. However, despite ongoing improvements in treatment technology, the five-year survival rate for advanced CRC continues to be low. The uncertainty of treatment effects and the limitations of options pose challenges to both doctors and patients. The current challenges facing CRC treatment include improving efficacy, reducing side effects and achieving personalized medicine. Insufficient understanding of CRC biomarkers hinders the selection of precise treatment pathways. In addition, the complex tumor microenvironment of CRC makes it difficult to overcome cell resistance and immune escape (41).

In recent years, researchers have begun to focus on the role of TIIC in CRC. The TIIC signature score serves as a novel instrument that utilizes single-cell RNA sequencing data to measure the presence and activity levels of these cells (42). This score helps identify patients who may respond to immunotherapy and provides a new perspective for understanding CRC. TIIC signature score has been applied in multiple clinical studies, especially in predicting the effect of immunotherapy, showing promise, such as in the study of breast cancer, lung cancer and other solid tumors, it can effectively distinguish patients with different prognoses (43).

In this study, we successfully identified CRC-related RNAs (TIIC-RNAs) specifically expressed in the immune microenvironment at the single-cell level using scRNA-seq datasets. We screened seven immune cells from CRC cells and nine microenvironment cells for analysis, and finally identified 137 of the most valuable TIIC-RNAs. These RNAs not only serve as possible biomarkers for diagnosing CRC patients but may also play a role in the mechanisms behind CRC progression, offering fresh insights for a deeper comprehension of the tumor immune microenvironment. Subsequently, based on the TIIC-RNAs identified above, we constructed a prognostic model that has excellent predictive ability for OS in CRC patients. We screened through multiple machine learning algorithms, identified the most reliable model and validated it. The ROC curve demonstrated its excellent sensitivity and specificity, especially in predicting 1 to 5-year survival. The findings indicate that the TIIC signature score may serve as an independent prognostic marker and could potentially surpass the conventional clinical staging system, thus offering robust evidence for personalized medicine. In addition, our study showed that the TIIC signature score can not only more accurately reflect the severity of CRC patients, but also effectively distinguish patients with different risk levels. Compared with other published prognostic models, our model showed significant superiority, suggesting that our model may become an important part of future clinical practice. The noticeable difference in survival rates among the high-risk and low-risk groups emphasizes the importance of the TIIC signature score as an indicator of prognosis. This discovery holds considerable importance in directing clinical treatment choices and developing individualized treatment strategies.

To investigate the potential biological mechanisms, we additionally examined the patterns of gene expression in groups characterized by high and low TIIC signature scores. Our results demonstrated that the cohort with a low TIIC signature score revealed an upregulation of pathways related to immunity, while the cohort exhibiting a high TIIC signature score displayed enhanced activation of BMP signaling pathways, along with the regulation of growth factors and several additional pathways. This disparity may arise from the enhanced ability of tumor cells in the high TIIC signature score group to evade immune system surveillance, resulting in a poorer prognosis. At the same time, the GSEA results also revealed different gene expression patterns between the two groups, especially in vascular morphogenesis, tubular morphogenesis, and protein modification process regulation. The study examined the ability of the TIIC signature score to predict the effects of immunotherapy. Our results revealed a relationship between the TIIC signature score and the effectiveness of immunotherapy in different cancer types. Importantly, patients who had higher TIIC signature scores seemed to show a better response to anti-PD-1/PD-L1 treatments, providing a conceptual basis for identifying appropriate candidates for immunotherapy. Furthermore, analysis using the TIDE algorithm corroborated these findings, suggesting that diminished TIIC signature scores could correspond to reduced rates of immunotherapy response, which holds significant implications for refining immunotherapy strategies. By conducting GSVA analysis on metabolic pathways within the KEGG database, we discovered a notable correlation between TIIC signature scores and specific metabolic processes. Notably, pathways including fatty acid elongation, degradation, and arachidonic acid metabolism exhibited increased activity in the group with higher TIIC signature scores. These findings suggest that metabolic reprogramming may be an important aspect of CRC development, and also propose a new direction: the possibility of improving treatment effects by regulating specific metabolic pathways. This provides new ideas for future drug development and treatment methods. Although our GSVA analysis revealed significant correlations between the TIIC signature score and several key metabolic pathways (such as fatty acid elongation, degradation, arachidonic acid metabolism, and glycerophospholipid metabolism), it is important to note that these findings are currently hypothesis-generating. Further experimental investigations, both *in vitro* and *in vivo*, are necessary to validate the causal relationships and elucidate the underlying molecular mechanisms linking TIIC-related immune responses with metabolic reprogramming in colorectal cancer.

In conclusion, the assessment of SNV mutations in conjunction with variations in CNV revealed the genetic diversity that exists between the two groups defined by TIIC signature scores. Notably, the detection of frequently mutated genes like APC, TP53, and TTN, in addition to copy number alterations on chromosome 7, indicates that these variations could significantly contribute to the onset and progression of CRC. Moreover, the heightened chromosomal instability noted in the group with a high TIIC signature score indicates a higher degree of genomic instability in these patients, potentially elucidating the factors contributing to their poor prognosis.

This study also has some limitations. First, this study mainly relies on data from public databases for analysis, which may not fully represent the true situation of all CRC patients, and lacks direct verification of patients in specific populations or regions. Second, although we verified the prognostic value of the model through multiple machine learning algorithms, the actual clinical application effect of the model still needs to be further confirmed through clinical trials. Furthermore, the precise molecular pathways linking TIIC signature scores with metabolic traits and responses to immunotherapy have yet to be completely clarified, necessitating additional experimental research in the future to corroborate these results. Finally, due to the limitations of sample size and data type, our analysis may not fully cover all relevant variables, so the impact of these factors should be carefully considered when interpreting the results.

In summary, this study not only deepened the understanding of CRC and its immune microenvironment, but also developed a prognostic model with potential clinical application value. More importantly, our work provides multiple new research directions for future research, including but not limited to exploring new therapeutic targets, optimizing immunotherapy strategies, and understanding tumor metabolic remodeling. Our research results are expected to promote progress in the diagnosis and treatment of CRC to improve the quality of life and survival rate of patients.

## 5 Conclusion

This research highlights the promise of the TIIC feature score for forecasting the prognosis of patients with CRC and suggests its utility in predicting immunotherapy outcomes while providing greater insights into the intricate nature of the tumor microenvironment. Subsequent studies should focus on evaluating the potential of TIIC feature score in clinical applications and verifying its applicability in diverse populations and different treatment scenarios.

## Data Availability

The public data in this study has been stored in a repository with the following access numbers: RNA expression profile and corresponding clinical data of TCGA (n=606): https://www.cancer.gov/ccg/research/genome-sequencing/tcga. GSE12945 (n=62): https://www.ncbi.nlm.nih.gov/geo/query/acc.cgi?acc=GSE12945, GSE17537 (n=55): https://www.ncbi.nlm.nih.gov/geo/query/acc.cgi?acc=GSE17537, GSE39582 (n=579): https://www.ncbi.nlm.nih.gov/geo/query/acc.cgi?acc=GSE39582. The experimental data is stored in Nut Cloud and can be downloaded at the address: https://www.jianguoyun.com/p/DWKg5L4Q-My3DRi8tfcFIAA.

## Supplementary material

Supplementary Figure 1 Validation of AIP, HNRNPH1, UBE2D2, and NFKB2 expression in CRC Cell Lines (LoVo and SW480) and Normal Colonic Epithelial Cell Line (NCM460) by qPCR.
