# Supplementary figures and images for: Tumor-infiltrating immune cell signature score reveals prognostic biomarkers and therapeutic targets for colorectal cancer
Source: Front Immunol. 2025 May 14;16:1583327. doi: 10.3389/fimmu.2025.1583327 (PMC12117586; doi:10.3389/fimmu.2025.1583327)

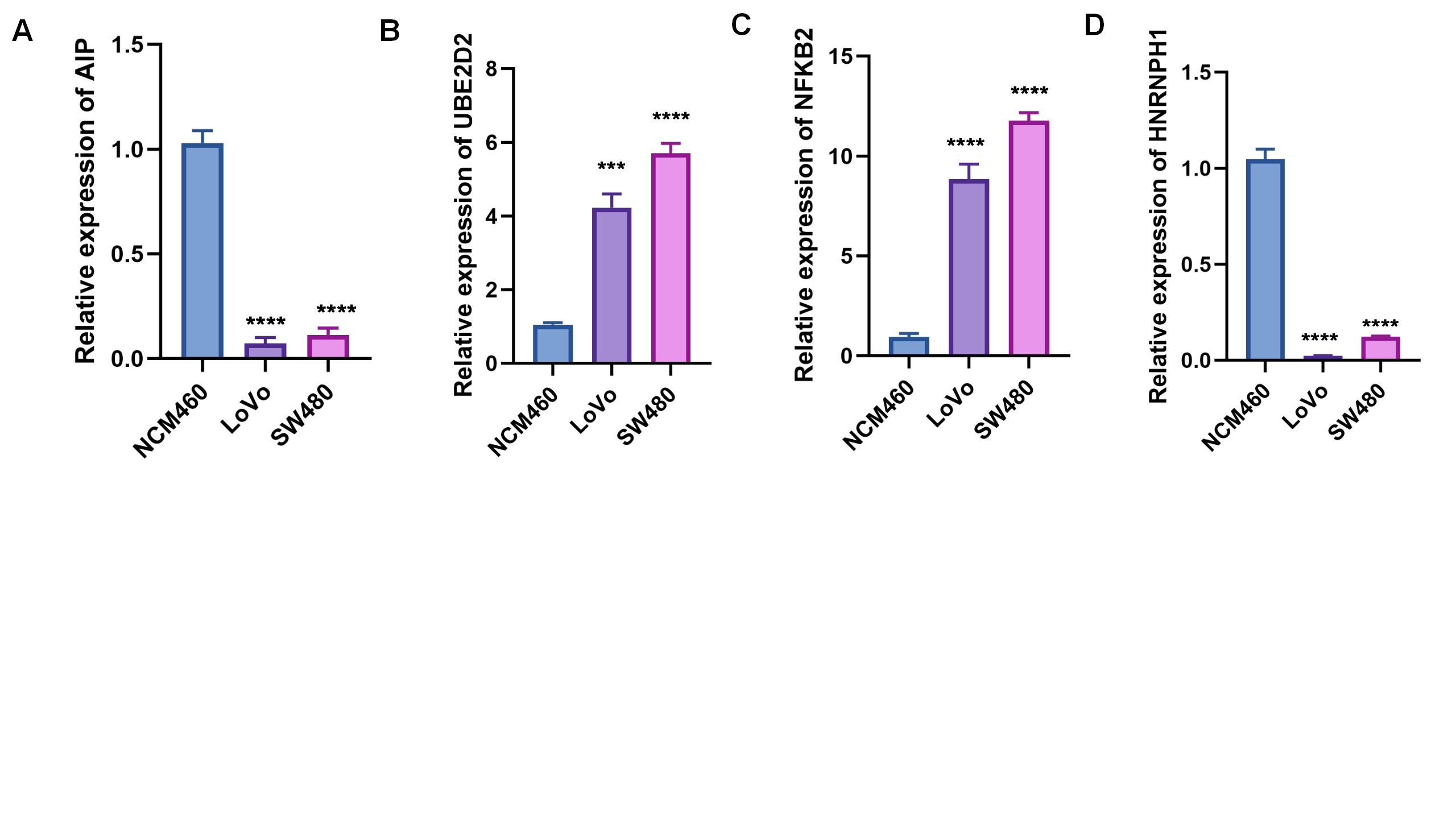

Supplement: Supplementary Figure 1 — Validation of AIP, HNRNPH1, UBE2D2, and NFKB2 expression in CRC Cell Lines (LoVo and SW480) and Normal Colonic Epithelial Cell Line (NCM460) by qPCR. [file Image1.png]
