# Supplementary material for: Tumor-infiltrating immune cell signature score reveals prognostic biomarkers and therapeutic targets for colorectal cancer
Source: Front Immunol. 2025 May 14;16:1583327. doi: 10.3389/fimmu.2025.1583327 (PMC12117586; doi:10.3389/fimmu.2025.1583327)
Supplement: Supplementary file 2 [file Table1.docx]

**Supplementary table 1. A list of primers used in this study.**

| Gene | Forward sequence (5’ to 3’) | Reverse sequence (5’ to 3’) |
| --- | --- | --- |
| GAPDH | GGAGCGAGATCCCTCCAAAAT | GGCTGTTGTCATACTTCTCATGG |
| AIP | TCCAAAAACGTGTGATACAGGAA | CTCCCCTTCTCGCATGGTG |
| UBE2D2 | TGGCAAGCTACAATAATGGGG | AAGGGGTAATCTGTTGGGAAATG |
| HNRNPH1 | ATTCAAAATGGGGCTCAAGGTAT | GTGTCAGGACTATTTGGACCAG |
| NFKB2 | ATGGAGAGTTGCTACAACCCA | CTGTTCCACGATCACCAGGTA |
